# Ubiquitin-specific Peptidase 42 (USP42) Functions to Deubiquitylate Histones and Regulate Transcriptional Activity*

**DOI:** 10.1074/jbc.M114.589267

**Published:** 2014-10-21

**Authors:** Andreas K. Hock, Arnaud M. Vigneron, Karen H. Vousden

**Affiliations:** From the Cancer Research UK Beatson Institute, Glasgow G61 1BD, Scotland, United Kingdom

**Keywords:** Chromatin, Deubiquitylation (Deubiquitination), Histone, RNA Polymerase II, Transcription, USP42

## Abstract

Ubiquitin-specific peptidase 42 (USP42) is a deubiquitylating enzyme that can target p53 and contribute to the stabilization of p53 in response to stress. We now show that USP42 can also regulate transcription independently of p53. USP42 co-localized with RNA polymerase II (RNA Pol II) in nuclear foci, bound to histone H2B, and deubiquitylated H2B. Depletion of USP42 increased H2B ubiquitylation at a model promoter and decreased both basal and induced transcription from a number of promoters. These results are consistent with a role for USP42 in regulating transcription by deubiquitylating histones.

## Teaser

**Background:** Ubiquitin modification of histones regulates gene expression.

**Results:** USP42 targets histone H2B at promoters, leading to decreased ubiquitylation. This correlates with the regulation of transcription driven by a number of transcription factors.

**Conclusion:** USP42 contributes to the modulation of transcription.

**Significance:** The identification of histone H2B as a target for USP42 extends our understanding of the factors that can regulate gene expression.

## Introduction

Post-translational modifications are critical mechanisms through which protein activity is regulated. Conjugation of ubiquitin can control the function of target proteins in multiple ways, including degradation, subcellular localization, and activity (1, 2). The consequences of ubiquitylation vary depending on the type and length of the ubiquitin chain, and many proteins are controlled through ubiquitylation (1–4). The process itself is highly regulated with a cascade involving ubiquitin-activating, ubiquitin-conjugating, and ubiquitin-ligating enzymes promoting the conjugation of ubiquitin to selected target proteins. This process can be reversed by the activity of deubiquitylating enzymes (DUBs)^4^ that remove ubiquitin (5, 6).

p53 is a potent tumor suppressor protein that is primarily regulated at the level of protein stability (7). A number of ubiquitin ligases that target p53 for polyubiquitylation and degradation have been described, including MDM2 (8, 9), Chip (10), Pirh2 (11), and ARF-BP1 (12). MDM2 itself is also regulated by ubiquitylation and degradation, and several DUBs, including USP7 (13), USP2a (14), and USP15 (15), have been shown to promote p53 degradation by deubiquitylating and stabilizing MDM2. p53 is also the direct target of a number of DUBs, including USP10 (16), USP29 (17), USP42 (18), and Otubain1 (19). After DNA damage and its phosphorylation by ATM, USP10 localizes to the nucleus to stabilize p53. USP42 was shown to play a role in accelerating the stabilization of p53 in response to genotoxic stress. However, it was clear from these studies that lack of USP42 delayed but did not prevent the full stabilization of p53 protein (18).

As with other protein modification systems, the potential number of target proteins exceeds the number of DUBs (around 100 in humans (20)), indicating that each DUB is likely to target many different proteins. In our continued analysis of USP42 function, we have identified monoubiquitylated histone H2B as a target for deubiquitylation by USP42. The dynamic interchange and balance between ubiquitylation and deubiquitylation of histones is critical for the regulation of transcription (21–26), and our data suggest that USP42 may be an important component of this fundamental level of control of transcriptional activity.

## EXPERIMENTAL PROCEDURES

### 

#### 

##### Tissue Culture

Cells were cultured in DMEM supplemented with glutamine and 10% FCS.

##### Plasmids

GFP-FLAG-USP42 and USP42 C120A have been described before (18). All deletion constructs were derived by PCR-based deletion using KOD Hot Start polymerase (Merck Millipore, 71842).

##### Transfection

Cells were transfected with the indicated plasmids (Genejuice, Merck Millipore, 70967) or siRNA (Hiperfect, Qiagen, 301705) as described before (18).

##### Western Blotting

Gels were transferred to a nitrocellulose membrane using a Mighty Small chamber from Hoefer. Blots were blocked in 5% milk TBS-Tween for at least 20 min and incubated with the appropriate antibodies overnight at 4 °C on a shaker. After three 5-min washes with TBS-Tween, the blots were incubated with the appropriate LI-COR Biosciences secondary antibody (in 5% milk TBS-Tween in a 1:10,000–20,000 dilution) followed by LI-COR Biosciences Odyssey detection (*K* set to 1). Antibodies used were: USP42 (Atlas, HPA006752), GFP (Abcam, ab6556; Roche Applied Science, 11814460001), H2B (Cell Signaling Technology, 2934), RNA Pol II (Santa Cruz, SC-65884), actin (Santa Cruz, SC-1616), tubulin (Santa Cruz, SC-8035), Ubi-H2B (Cell Signaling Technology, 5546), H2A (Cell Signaling Technology, 3636), and Ubi-H2A (Millipore, 05-678; Cell Signaling Technology, 8240). Western blots were quantified using the LI-COR Biosciences Image Studio software V 2.1.10 and plotted using PRISM software from GraphPad.

##### Immunoprecipitation

Cells were washed once with PBS, scraped in 1 ml of PBS into a 1.5-ml Eppendorf tube, and centrifuged for 5 min at 3000 rpm at 4 °C in a refrigerated Eppendorf microcentrifuge. Cells were subsequently lysed in lysis buffer (50 mm Tris-HCl, pH 7.4, 150 mm NaCl, 0.25% deoxycholic acid, 1% Nonidet P-40, protease inhibitor mixture (Roche Applied Science, 04693159001)) and sonicated in a Bioruptor (20 s at lowest setting) to lyse the chromatin. Magnetic protein G beads (Invitrogen, 10004D) or Dynabeads Rat anti-Mouse IgM (Invitrogen, 11039D) were washed three times in lysis buffer and blocked in lysis buffer plus 5% BSA for 1 h. Beads, antibody, and lysates were mixed; the volume was topped up to 700 μl with lysis buffer and samples were rotated overnight at 4 °C, washed three times in lysis buffer and boiled in 1× SDS reducing loading buffer for elution.

##### Immunofluorescence

Confocal immunofluorescence was performed as described previously (27). In brief, cells were cultured on coverslips at approximately 70% confluence. At harvesting, cells were washed three times in PBS, fixed in 4% paraformaldehyde for 15 min, and stained with DAPI solution. For colocalization of USP42 and DNA-bound RNA Pol II, cells were washed in CSK buffer (0.3 m sucrose, 10 mm PIPES, 3 mm MgCl_2_, 1 mm EGTA, 0.5% Triton X-100) prior to fixation to remove unbound soluble proteins.

##### FACS Analysis

A U2OS clone stably expressing CMV Cherry and doxycycline-inducible GFP was established by puromycin selection over 3 months followed by colony picking and characterization. After transfection with plasmids or siRNA, cells were induced with doxycycline or solvent control as described in the text. Cells were then harvested by trypsinization and resuspended in 1% FCS in PBS-Tween followed by immediate FACS analysis (BD FACSAria). FlowJo was used to determine the median fluorescence.

##### Chromatin Immunoprecipitation

Chromatin immunoprecipitation was performed as described before (18).

##### In Vitro Deubiquitylation Assays

USP42 constructs were expressed in HEK293T cells, lysed in radioimmune precipitation assay buffer, and purified by precipitation using GFP-trap_M matrix (CromoTek, gtm-20). Histones were purified from HeLa cells using an acid extraction protocol (Abcam). In brief, nuclear extracts were incubated with 0.2 n HCl overnight followed by centrifugation, aliquoting, freezing in liquid N_2_, and storage at −80 °C. Assays: HeLa histones were thawed, rebuffered with NaOH, and diluted in 50 mm Tris, pH 8 to a volume of 80 μl. Aliquots were added to purified USP42 on GFP-trap_M matrix and incubated at room temperature, and 10-μl samples were taken at the indicated time points, mixed 1:1 in 2× SDS loading buffer, and boiled for 5 min to stop the deubiquitylation reaction.

##### Fractionation

Cells were treated as described previously (31). In brief, after fractionation into nuclear and cytoplasmic fractions, the nuclear fraction was further separated by incubation in lysis buffer (described above) and centrifuged into DNA bound (pellet) and nuclear soluble fractions.

## RESULTS AND DISCUSSION

**FIGURE 1.**
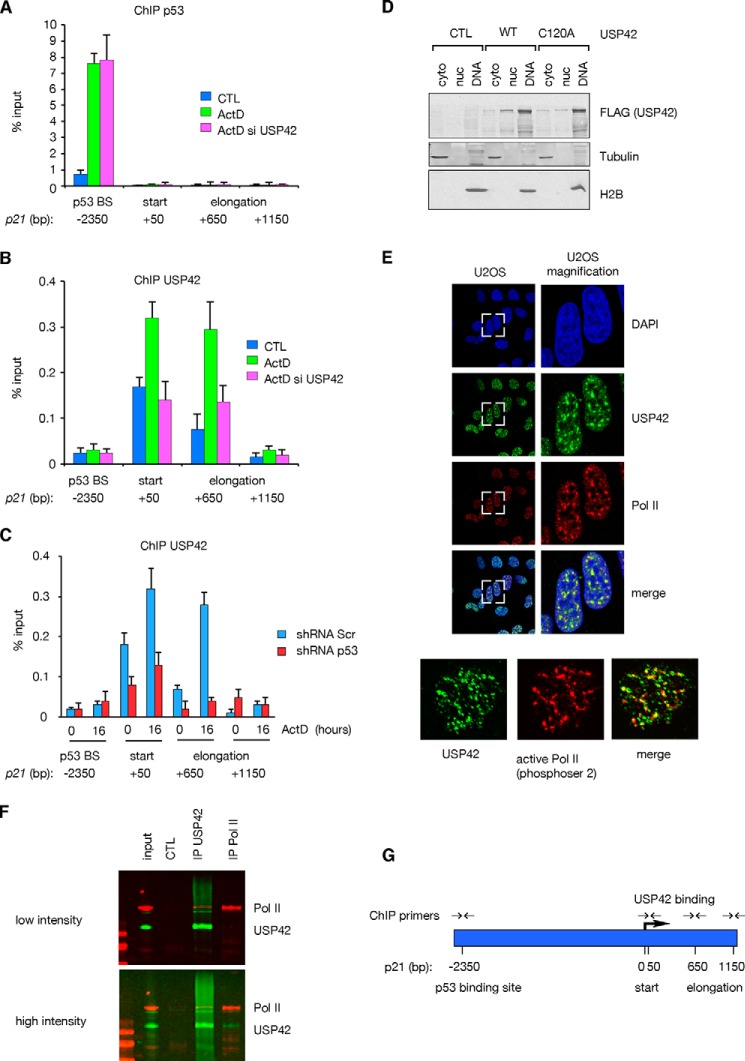
**USP42 binds to the start region of transcriptionally active *p21* promoter and localizes in nuclear foci.** *A*, chromatin immunoprecipitation showing the recruitment of p53 to its binding site and not to the start and elongation region of the *p21* gene upon its induction. *Error bars* represent the S.D. of three independent replicas. *B*, chromatin immunoprecipitation showing the recruitment of USP42 to the start and elongation region of the *p21* gene and not to the p53 binding site. *Error bars* represent the S.D. of three independent replicas. *C*, chromatin immunoprecipitation showing the recruitment of USP42 to the *p21* start and elongation region in control and shp53 cells with and without actinomycin D (*ActD*) treatment. *Error bars* represent the S.D. of three independent replicas. *D*, fractionation of U2OS cells overexpressing USP42 WT and C120A. USP42 associates with the chromatin independently of its catalytic activity. *E*, confocal immunofluorescence visualizing the localization of endogenous USP42 and DNA-bound RNA Pol II (total and activated) after extracting soluble proteins in U2OS cells. *F*, endogenous coimmunoprecipitation of USP42 and RNA Pol II. U2OS cells were lysed, and the proteins were immunoprecipitated with the indicated antibodies. *G*, map of the *p21* locus with primer amplicons. Positions are relative to the start site. *CTL*, control; *cyto*, cytoplasmic; *nuc*, nuclear; Pol II, RNA Pol II; *Scr*, scrambled.

Our previous work showed that USP42 can target p53 for deubiquitylation, with depletion of USP42 resulting in delays in stabilization of p53 and recruitment of p53 to target gene promoters (18). However, these studies also showed that USP42 loss did not impact the fully activated levels of p53, which were stabilized to a similar extent irrespective of USP42 expression within 16 h of genotoxic or ribosomal stress. In these studies, the low dose of actinomycin D used has been shown to induce ribosomal stress and specifically activate p53 rather than more generally interfere with transcription (28). In agreement with these published data, we have found that depletion of USP42 does not impact recruitment of p53 to the *p21(CDKN1A)* promoter when examined at a 16-h time point after induction of a p53 response with low dose actinomycin D (Fig. 1*A*). Interestingly, USP42 was also recruited to the *p21* locus after actinomycin D treatment, but importantly the binding of USP42 was at sites distinct from those bound by p53. Although ChIP assays detected p53 on its well characterized binding site in the *p21* distal promoter (29), USP42 was detected further downstream at the transcription start site of the *p21* gene (Fig. 1*B*). To determine whether USP42 recruitment depends on the activation of *p21* transcription by p53, we repeated the experiment in control and p53 knockdown cells (Fig. 1*C*). Although USP42 recruitment to the start site and the first intron was clearly increased following low dose actinomycin D treatment, this was at least partially dependent on p53 because a knockdown of p53 caused a reduction of USP42 recruitment. Taken together these data suggest that USP42 participates in transcription regulation through a mechanism that is dependent on p53 but likely to be independent of direct binding to p53. An overview of the ChIP primers is shown in Fig. 1*G*.

To further understand this role of USP42, we examined its localization in the cell. Fractionation studies indicated that USP42 was associated with the insoluble chromatin fraction, and this localization was independent of DUB activity because it was also seen with the catalytically inactive USP42 mutant C120A (Fig. 1*D*). Immunofluorescence analysis of endogenous proteins showed that USP42 was present in nuclear foci that co-localized with DNA-bound RNA Pol II using antibodies to detect both total and activated (phosphorylated) RNA Pol II (Fig. 1*E*), and endogenous USP42 and RNA Pol II were shown to co-immunoprecipitate (Fig. 1*F*). This was of particular interest because a proteomic analysis of USP42-binding proteins had shown association of USP42 with histone H2B and other components of the transcriptional machinery (not shown).

**FIGURE 2.**
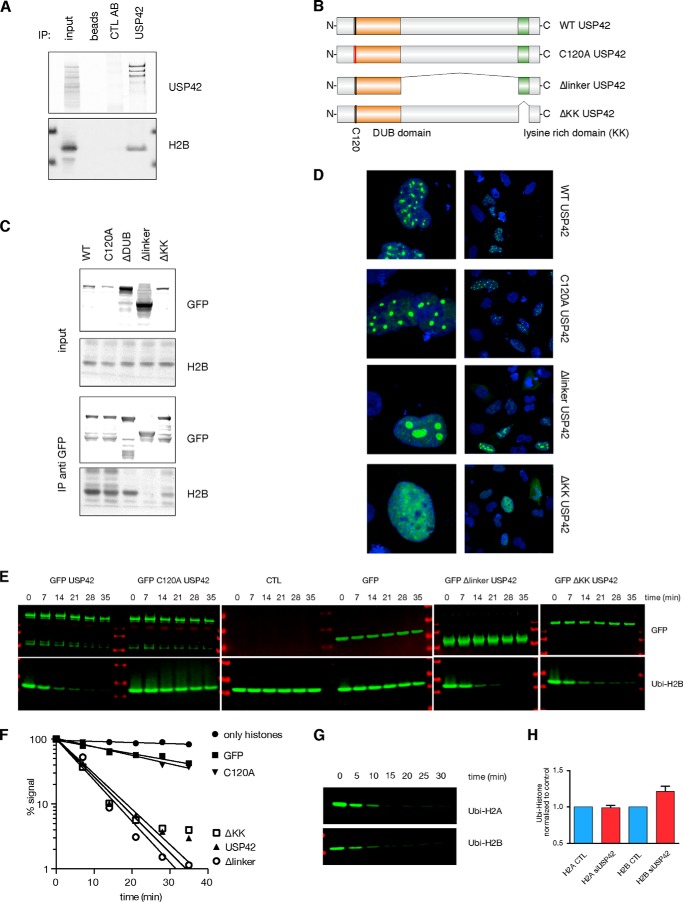
**The linker and lysine-rich domain of USP42 are necessary for appropriate localization of USP42 and interaction of histone H2B.** *A*, endogenous coimmunoprecipitation showing specific interaction of USP42 and H2B. *B*, schematic representation of the USP42 overexpression mutants used in subsequent experiments. *C*, immunoprecipitation of GFP-USP42 mutants to determine their interaction with H2B. Although the catalytic activity of USP42 is not required to bind H2B, deletion of the linker domain causes loss of H2B association, and deletion of the lysine-rich domain leads to a reduction of interaction. *D*, confocal immunofluorescence visualizing the localization of GFP-USP42 wild type and mutant proteins. Although the catalytic activity of USP42 does not alter the localization of USP42, deletion of the linker domain causes accumulation in the nucleoli, and deletion of the lysine-rich domain leads to a diffuse nuclear localization. *E*, *in vitro* deubiquitylation assay of Ubi-H2B by USP42. Wild type USP42 efficiently deubiquitylates Ubi-H2B. Disruption of the DUB domain renders USP42 inactive toward Ubi-H2B. ΔKK mutant and Δlinker mutants, which both harbor an intact DUB domain, retain the ability to deubiquitylate histone H2B. *F*, quantification of *E*. Ubi-H2B Western blots shown in *E* were quantified using the LI-COR Biosciences Odyssey. *G, In vitro* deubiquitylation assay of Ubi-H2B and Ubi-H2A by USP42. *H*, depletion of USP42 leads to an increase in H2B ubiquitylation and does not alter H2A ubiquitination. LI-COR quantification showing levels of H2B and H2A ubiquitylation (*Ubi-Histone*) normalized to unmodified histone with and without USP42 knockdown. *Error bars* represent the S.D. of three independent replicas. *IP*, immunoprecipitation; *CTL*, control.

To confirm this interaction, we immunoprecipitated endogenous USP42 from cells and were able to detect a specific interaction with histone H2B (Fig. 2*A*). Expression of USP42 mutants targeting the linker domain and the C-terminal lysine-rich domain (Fig. 2*B*) showed that although the DUB-inactive USP42 C120A mutant retained the ability to bind histone H2B, this interaction was reduced with the ΔKK mutant and lost with the Δlinker mutant (Fig. 2*C*). Immunofluorescence studies confirmed that both wild type and C120A USP42 proteins retained localization within nuclear foci previously associated with DNA-bound RNA Pol II, whereas the ΔKK mutant showed a diffuse nuclear localization, and the Δlinker mutant was relocalized to subnuclear structures likely to be nucleoli (Fig. 2*D*).

The binding of USP42 to histone H2B suggested that USP42 may target histones for deubiquitylation. Indeed, *in vitro* analysis showed that USP42 is able to efficiently deubiquitylate histone H2B (Fig. 2, *E* and *F*). This activity was dependent on the DUB activity of USP42 because the C120A mutant was unable to deubiquitylate histone H2B in this assay (Fig. 2, *E* and *F*). Interestingly, the ΔKK mutant and Δlinker mutants retained the ability to deubiquitylate histone H2B in this *in vitro* assay, suggesting that the failure of these mutants to effectively target histone H2B in cells is a reflection of their inappropriate localization rather than a loss of DUB activity. In the *in vitro* assay, USP42 showed a similar ability to deubiquitylate histones H2A and H2B (Fig. 2*G*). However, in cells, the ubiquitination of histone H2A was not affected by USP42 depletion, whereas the overall level of ubiquitylated H2B was modestly but consistently increased (Fig. 2*H*). These results indicate that the ability of USP42 to target proteins is regulated by factors in addition to DUB activity such as the control of appropriate cellular localization.

**FIGURE 3.**
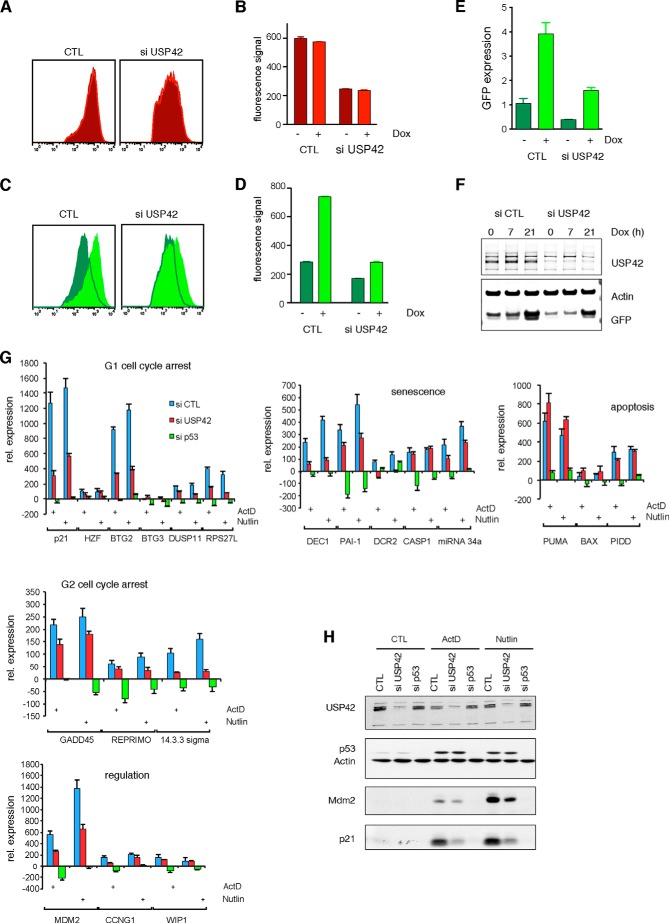
**USP42 is necessary for efficient transcription of reporter genes.** *A–D*, FACS analyses of a U2OS clone stably expressing Cherry and doxycycline-inducible GFP. USP42 knockdown leads to a reduction of Cherry signal (*A* and *B*) and to a drop of GFP signal in both the basal and induced transcription (*C* and *D*). *Error bars* represent the S.D. of three technical replicas. *E*, quantitative RT-PCR analysis of GFP expression. The U2OS clone was induced with doxycycline (*Dox*) with and without USP42 knockdown, and the GFP mRNA was quantified by real time PCR. GFP mRNA levels dropped after USP42 knockdown both in basal conditions and after GFP induction by doxycycline. *Error bars* represent the S.E. of three technical replicas. *F*, Western blot for GFP and USP42 visualizing the reduction of USP42 protein after knockdown and the resulting drop in GFP protein levels in basal and doxycycline-induced conditions. *G*, mRNA expression analysis of the indicated p53 target genes by quantitative RT-PCR. Knockdown of USP42 decreases mRNA expression of most p53 target genes. Knockdown of p53 was used to confirm p53-specific activation of promoters. *Error bars* represent the S.D. of three independent replicas. *H*, protein expression analysis of the indicated p53 target genes by Western blot. Knockdown of USP42 decreases protein expression of p53 target genes. Knockdown of p53 was used to confirm p53-specific activation. *CTL*, control; *ActD*, actinomycin D; *rel.*, relative.

Because histone H2B ubiquitylation and deubiquitylation are important for transcriptional regulation (22), we postulated that USP42 may be able to influence transcription efficiency from promoters beyond those regulated by p53. To analyze an effect of USP42 on more general transcription, we turned to a model system in which fluorescent reporter proteins (Cherry and GFP) are stably integrated in the genome and expressed from constitutive (CMV, Cherry) or inducible (doxycycline, GFP) promoters. This system makes it possible to directly manipulate the induction of GFP without having to take other regulatory processes into account. In addition, this system allows us to normalize expression of the reporter protein on a per cell basis rather than to a housekeeping factor that itself may be regulated by USP42. siRNA depletion of USP42 resulted in an overall reduction of CMV-driven Cherry expression detected directly by FACS (Fig. 3, *A* and *B*). Interestingly, depletion of USP42 also slightly reduced the basal expression of doxycycline-driven GFP and substantially decreased the induced levels of GFP expression following doxycycline treatment of the cells (Fig. 3, *C* and *D*). The inhibition of GFP expression on a basal level and postinduction with doxycycline could be observed at both mRNA (Fig. 3*E*) and protein levels (Fig. 3*F*), confirming that modulation of USP42 expression regulates the transcription of both constitutive and inducible promoters.

To determine whether the knockdown of USP42 also affects the transcription of endogenous loci, we analyzed the effect of USP42 knockdown on the transcription of p53 target genes upon its induction with actinomycin D and Nutlin. *p21* was chosen because we have demonstrated that USP42 is recruited to the *p21* transcription start site after p53 induction (Fig. 1*B*), and induction of p53 itself relies mainly on stabilization of protein levels rather than induction of p53 transcription. To extend the study, we also examined a further 19 p53-inducible genes (Fig. 3*G*). As expected, p53 target genes were induced upon both Nutlin and actinomycin D treatment, resulting in increased mRNA expression (Fig. 3*G*). USP42 depletion decreased the transcription of most of these target genes, although some (*CASP1*, *PUMA*, *BAX*, *PIDD*, and *WIP1*) were not affected (Fig. 3*G*). We have shown previously that USP42 depletion retards the stabilization of p53. Importantly, therefore, at this time point (16 h), p53 protein was fully stabilized regardless of USP42 status (Fig. 3*H*). Nevertheless, expression of target proteins p21 and MDM2 remained at lower levels, correlating with the lower mRNA expression (Fig. 3*G*).

**FIGURE 4.**
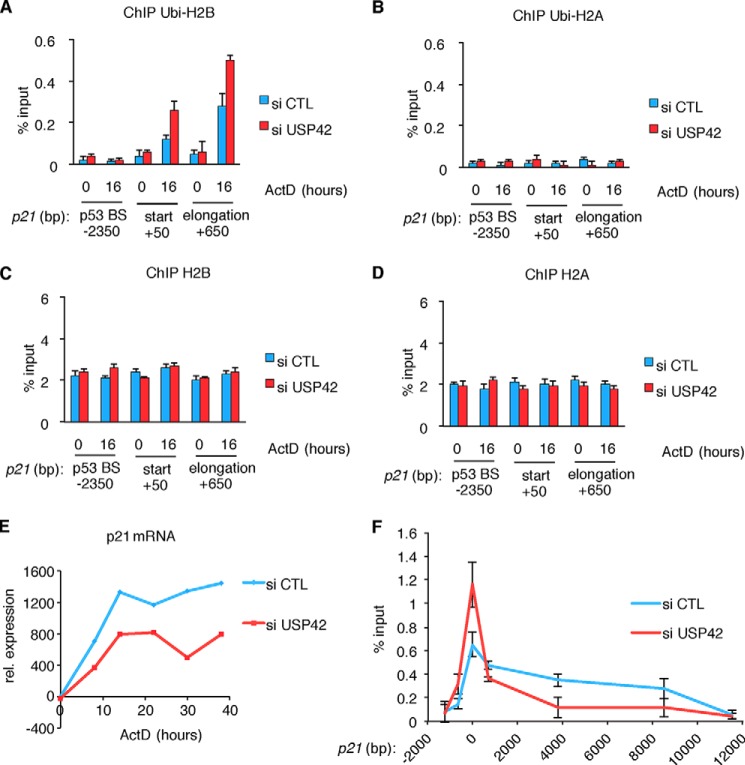
**USP42 knockdown increases endogenous H2B ubiquitylation specifically on the start and early extension sites of the *p21* promoter upon its induction.** *A–D*, chromatin immunoprecipitations showing the ubiquitylation status of H2B and H2A on the *p21* gene following USP42 knockdown. H2B ubiquitylation (*ubi-H2B*) increases upon *p21* induction at the initiator and the first intron. This is further increased by knockdown of USP42 (*A*), whereas H2A ubiquitylation (*ubi-H2A*) is not influenced by *p21* induction or USP42 knockdown (*B*). The difference in ubiquitylation is not an indirect result of general histone deposition because H2B and H2A levels are not altered (*C* and *D*). *Error bars* represent the S.D. of three independent replicas. *E*, expression analysis of *p21* mRNA by quantitative RT-PCR. Knockdown of USP42 decreases *p21* mRNA up to 38 h. *F*, chromatin immunoprecipitation showing the recruitment of RNA Pol II to the *p21* gene upon p53 induction. USP42 knockdown (*red*) increases RNA Pol II levels closer to the start site of transcription, whereas a reduced amount of RNA Pol II is observed in the distal part of the gene relative to control (*blue*). *Error bars* represent the S.D. of three independent replicas. *ActD*, actinomycin D; *CTL*, control; *rel.*, relative; *p53BS*, p53 binding site.

To assess how the knockdown of USP42 induces a change in transcription from an endogenous promoter, we investigated the influence of USP42 reduction on the *p21* promoter. Although p53 was recruited to the p53 response element following actinomycin D treatment (Fig. 1*A*), there was no change in the level of H2B ubiquitylation around this site, and the knockdown of USP42 did not lead to an increase of H2B ubiquitylation here (Fig. 4*A*). By contrast, increased H2B ubiquitylation was detected at the transcriptional start site and first intron of the *p21* gene following actinomycin D treatment (where USP42 was bound; Fig. 1*B*). Reduction of USP42 expression by siRNA led to a further increase of H2B ubiquitylation (Fig. 4*A*) but did not alter overall deposition of histone H2B (Fig. 4*C*). Interestingly, USP42 did not affect H2A ubiquitylation (Fig. 4*B*) or deposition (Fig. 4*D*). These results suggest that USP42 can specifically modulate the levels of H2B ubiquitylation in the first nucleosomes of the *p21* coding sequences in response to p53 activation. To determine the outcome of USP42 knockdown on *p21* transcription, we analyzed p21 mRNA levels at several time points after induction with and without USP42 depletion (Fig. 4*E*). As we had demonstrated before, USP42 knockdown lowers p21 transcription at the early time points of p53 induction as a reflection of the reduced levels of p53 (18). However, at time points where p53 levels (Fig. 3*H*) and p53 recruitment to the *p21* promoter (Fig. 1*A*) have become equal, the levels of *p21* mRNA expression remain lower in USP42-depleted cells (Fig. 4*E*). To determine whether the increased levels of Ubi-H2B observed at the *p21* locus after USP42 depletion affected RNA Pol II migration, we analyzed RNA Pol II distribution in a ChIP experiment. Interestingly, we could observe increased levels of RNA Pol II association at the proximal region of the *p21* transcriptional start site upon USP42 knockdown, whereas USP42-depleted cells showed a reduction in RNA Pol II levels in the more distal part of the *p21* gene (Fig. 4*F*). Taken together these results are consistent with a model where stalling of RNA Pol II at the transcriptional start site and inhibition of transcriptional progression are caused by an inability to deubiquitinate H2B.

**FIGURE 5.**
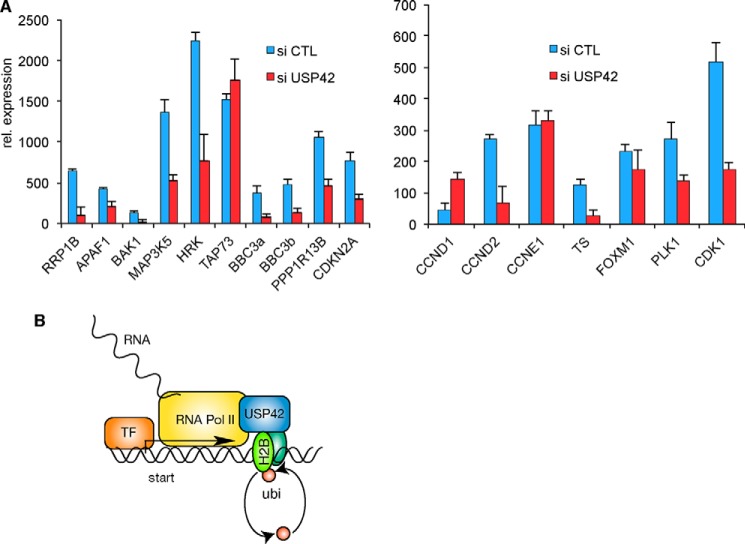
**USP42 is necessary for efficient transcription of E2F1 target genes.** *A*, mRNA expression analysis of indicated E2F1 target genes by quantitative RT-PCR. Knockdown of USP42 decreases mRNA expression of E2F1 target genes. *Error bars* represent the S.D. of three independent replicas. *B*, model of proposed mechanism for how the deubiquitylation activity of USP42 influences transcription. *CTL*, control; *rel.*, relative; *TF*, transcription factor.

To extend our studies beyond p53, we investigated how USP42 reduction alters the induction of E2F1 target genes in an inducible system. As observed with the induction of GFP (Fig. 3) and p53 (Figs. 3 and 4), depletion of USP42 reduced the mRNA expression of most of the E2F1-induced target genes examined (Fig. 5*A*). Again, we found three examples of genes that were not significantly affected by USP42 depletion (*TAp73*, *FOXM1*, and *CCNE1*), indicating that the requirement for USP42 to promote transcription is not universal.

Taken together the results support a model whereby USP42 directly influences transcription by deubiquitylating histone H2B at transcriptional start sites (Fig. 5*B*). As demonstrated in Fig. 4, the effect of USP42 is specific to H2B at the transcriptional start site (correlating with the site of USP42 binding to the promoter) rather than more globally affecting histone ubiquitylation. This correlates with our observation that USP42 depletion has only a modest but reproducible impact on overall ubiquitylated H2B levels (Fig. 2*H*). We suggest that this may reflect the selectivity of USP42 function in only controlling ubiquitylation, and thereby transcription, of a subset of promoters and the fact that promoters only constitute a very small part of the genome. How USP42 gets recruited to the promoters is not yet understood, although our data suggest that efficient recruitment of USP42 depends on the binding of the transcription factors such as p53 (Fig. 1*C*).

Our study identifies a role for USP42 in the regulation of transcription and provides evidence that this may be mediated through the control of histone ubiquitylation. A previous proteomic analysis of the DUB interactome described an association of USP42 with histones, but also with a large number of other proteins, including other DUBs (30). Although we can identify an effect of USP42 on histone H2B ubiquitylation, it is clear that the biological outcome of USP42 activity may also reflect the interaction with many other proteins. Although ubiquitylation of histones is an important mechanism to regulate transcription, the exact outcome of the regulation or misregulation of histone ubiquitylation is complex, making the effect of USP42 activity difficult to predict. This activity of USP42 acts in concert with our previously described function in the transient regulation of p53 stability, and we believe these to be independent functions of USP42. How USP42 is recruited to the transcriptional start site of *p21* will require further investigation, although our work suggests that this does not depend on DUB activity. Possibly USP42 is a component of the transcriptional machinery that is recruited in response to p53 binding to the promoter. Our preliminary studies suggest that USP42 modulation will have disparate effects on transcription depending on the promoter and activating signal, and future studies will be required to define in more detail the physiological function of USP42. However, our work reveals another component of this critical pathway for the regulation of gene expression in mammalian cells.

## Footnotes

4 The abbreviations used are:

DUB: deubiquitylating enzyme
ActD: actinomycin D
ChIP: chromatin immunoprecipitation
USP: ubiquitin-specific peptidase
Ubi: ubiquitin
Pol: polymerase.
